# Treatment of antipsychotic-induced hyperprolactinemia: an umbrella review of systematic reviews and meta-analyses

**DOI:** 10.3389/fpsyt.2024.1337274

**Published:** 2024-03-05

**Authors:** Qitong Jiang, Tian Li, Lei Zhao, Yue Sun, Zhen Mao, Yujie Xing, Chuanyue Wang, Qijing Bo

**Affiliations:** ^1^The National Clinical Research Center for Mental Disorders & Beijing Key Laboratory of Mental Disorders & Beijing Institute for Brain Disorders Center of Schizophrenia, Beijing Anding Hospital, Capital Medical University, Beijing, China; ^2^Advanced Innovation Center for Human Brain Protection, Capital Medical University, Beijing, China

**Keywords:** antipsychotic, hyperprolactinemia, adverse effects, aripiprazole, metformin, dopamine agonists, umbrella review

## Abstract

**Background:**

Hyperprolactinemia is a common antipsychotic-induced adverse event in psychiatric patients, and the quality of clinical studies investigating the best treatments has varied. Thus, to better summarize the clinical evidence, we performed an umbrella review of overlapping systematic reviews and meta-analyses for the treatment of antipsychotic-induced hyperprolactinemia.

**Methods:**

The PubMed, Cochrane Library, PsycINFO, Scopus and EMBASE were searched, and reviews and meta-analyses meeting our inclusion criteria were selected. Relevant data were extracted, and an umbrella review was conducted of all included meta-analyses. The quality of included meta-analyses was assessed by using PRISMA scores and AMSTAR 2 quality evaluation. Finally, the clinical evidence for appropriate treatments was summarized and discussed.

**Results:**

Five meta-analyses published between 2013 and 2020 met the requirements for inclusion in this umbrella review. The PRISMA scores of the included meta-analyses ranged from 19.5–26. AMSTAR 2 quality evaluation showed that 2 of the 5 included meta-analyses were of low quality and 3 were of very low quality. The included meta-analyses provide clinical evidence that adding aripiprazole or a dopamine agonist can effectively and safely improve antipsychotic-induced hyperprolactinemia. Two meta-analyses also showed that adjunctive metformin can reduce serum prolactin level, but more clinical trials are needed to confirm this finding.

**Conclusion:**

Adjunctive dopamine agonists have been proven to be effective and safe for the treatment of antipsychotic-induced hyperprolactinemia. Among the researched treatments, adding aripiprazole may be the most appropriate.

## 1 Introduction

Schizophrenia is a chronic and disabling disease (1) for which antipsychotics are currently the main first-line treatment (2). Chemical neurotransmitters associated with schizophrenia include serotonin, norepinephrine, acetylcholine, dopamine, gamma-aminobutyric acid (GABA), and others. Among them, the dopamine system is predominant (3). Dopamine mediates activity through five G protein-coupled receptors, which are divided in two subgroups, D_1_-like receptors (D_1_ and D_5_) and D_2_-like receptors (D_2_, D_3_, and D_4_) (4). The occurrence of psychotic symptoms is closely related to the dopamine D_2_ receptor (5). Long-term clinical application of antipsychotics has established that drugs acting on the D_2_ receptor can significantly improve the positive symptoms of schizophrenia (6). Antipsychotics block the mesolimbic dopaminergic pathway and mesocortical dopaminergic pathway, inhibit neuronal activity, and also affect the nodular-funnel pathway of the hypothalamus, thereby leading to an increase in prolactin (7). Prolactin is synthesized and secreted by prolactin cells in the anterior pituitary gland, and this process can be inhibited by dopamine. In fact, any factors that reduce the action of dopamine on D_2_ receptors can lead to an increase in prolactin (8).

A continuously elevated prolactin level beyond the normal range for any reason is known as hyperprolactinemia (9). Hyperprolactinemia is one of the most common antipsychotic-induced adverse events in psychiatric patients, and it can lead to menstrual disorders, gynecomastia, and galactorrhea (10). The reported incidence ranges for antipsychotic-induced hyperprolactinemia were 18%–72% in men and 42%–93% in women (11–14). Previous studies have suggested that antipsychotic-induced hyperprolactinemia is associated with the long dissociation time-course of these drugs (15) and their relatively poor ability to cross the blood–brain barrier (BBB) (16). Improving antipsychotic-induced hyperprolactinemia can increase medication compliance among patients (17, 18). However, treatment of antipsychotic-induced hyperprolactinemia has been a challenge clinically (19). The commonly used methods to improve antipsychotic-induced hyperprolactinemia include reducing the doses of the antipsychotic, switching to another antipsychotic that has a lesser effect on prolactin, adding a dopamine agonist, adding aripiprazole, and adding metformin (20). Among these methods, research to date has mainly focused on the adjunctive use of aripiprazole.

Aripiprazole exhibits a unique receptor binding characteristic. It is a partial agonist for dopamine D_2_ and D_3_ receptors that inhibits dopamine activity when the dopamine level is high and stimulates dopamine activity when the dopamine level is low (21). As a result of this characteristic, aripiprazole can be a stabilizer of dopamine and serum prolactin levels. Dopamine receptor agonists, including bromocriptine, cabergoline, and others, are widely used to treat hyperprolactinemia due to any reason. Dopamine agonist therapy is indicated for all patients with menstrual disorders, osteoporosis, and other symptoms caused by hyperprolactinemia (8). Some studies have also reported the effectiveness of traditional Chinese medicine in treating hyperprolactinemia (22–24), but the relevant clinical evidence remains insufficient.

In recent years, several meta-analyses have been conducted to explore the best treatment strategy for hyperprolactinemia. However, the quality of these meta-analyses has varied, and their conclusions have included some inconsistencies. Therefore, the present study aimed to provide an umbrella review of overlapping systematic reviews and meta-analyses of treatments for antipsychotic-induced hyperprolactinemia to identify the best clinical evidence for treatment selection.

## 2 Methods

### 2.1 Inclusion and exclusion criteria

Articles were selected for analysis according to the following inclusion criteria: (1) meta-analysis or systematic review based on randomized controlled trials (RCTs) or observational studies; (2) participants were adults or adolescents with diagnosed psychotic disorders (schizophrenia, bipolar disorder with psychotic features, schizoaffective disorder, psychotic disorder not otherwise specified, etc.), without restrictions of gender, race and length of disease duration; (3) reporting of at least one outcome (serum prolactin level, prolactin-related symptoms, adverse events, etc.); and (4) published in English. Articles that meet the following criteria were excluded: (1) nonhuman subjects; (2) lack of necessary information; (3) network meta-analysis; (4) inappropriate comparison, outcome, study type or population (for example, studies focused on general population or mixed population were excluded); and (5) full-text not accessible.

### 2.2 Search strategy

Two researchers independently searched the PubMed, Cochrane Library, PsycINFO, Scopus and EMBASE. The literature searches were conducted since the inception of the databases up to November 2023. The key search terms included “antipsychotic induced hyperprolactinemia”, “treatment”, “meta analysis”, “meta-analysis”, and “systematic review”. A more specific example of a PubMed search is as follows: “antipsychotic”[Title/Abstract] AND “hyperprolactinemia”[Title/Abstract] AND (“meta analysis”[Title/Abstract] OR “meta-analysis”[Title/Abstract] OR “systematic review”[Title/Abstract]) AND “treatment”[Title/Abstract]. After elimination of duplicates and screening of the titles and abstracts, articles that met the inclusion criteria were selected. The full texts of the retained articles were downloaded and evaluated in detail. Citations were also screened manually to identify other potentially eligible articles.

### 2.3 Data extraction

All useful information and data were extracted from the selected studies by two authors independently and entered into a standard, simple form with repeated checking. The following types of data were collected for each meta-analysis: first author, types and number of included studies, publication year, Information on antipsychotic, population, intervention measures, outcomes, quality assessment tool, results and main conclusion.

### 2.4 Quality evaluation

A quality assessment plan was developed in advance with the research questions. The PRISMA Statement consists of 27 items and is widely used to evaluate the quality of meta-analyses and systematic reviews (25). A PRISMA score of 21–27 points reflects a high-quality study; 15–21 points indicates medium quality; and <15 points is considered as low quality. It primarily assesses whether the report is transparent, complete and accurate, irrespective of the soundness of the methodology. Relying solely on the PRISMA score for evaluation accurately gauges the author’s writing comprehension ability but falls short in assessing the quality of the review’s planning and conducting.

To address this limitation, the present umbrella review also employed The Assessment of Multiple Systematic Reviews 2 (AMSTAR 2) (26) scoring standard to assess the quality of the methodology. It comprehensively evaluates systematic reviews and meta-analyses from multiple aspects, such as the literature search, statistical analysis, bias and conflict of interest. It is the most commonly used tool to evaluate the methodological quality of meta-analyses internationally. AMSTAR 2 is applied to meta-analyses or systematic reviews based on RCTs and/or nonrandomized studies of interventions (NRSIs), but does not include network meta-analyses. For this reason, to ensure the consistency of quality evaluation, network meta-analyses were excluded from the present umbrella review.

## 3 Results

### 3.1 Research selection and characteristics

**Figure 1:**
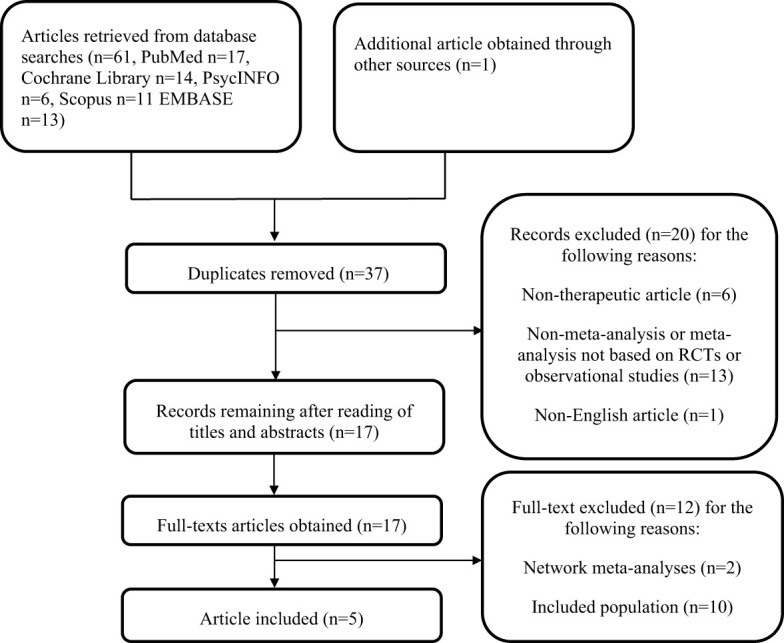
Flow diagram of article selection.

From our searches of three databases, 62 articles were retrieved. After the removal of duplicates, 37 articles remained. Based on screening of titles and abstract, 17 articles were selected for full-text evaluation. The full texts of the 17 potentially relevant articles were downloaded and assessed completely. Finally, 5 meta-analyses (27–31) that met the requirements were included in this systematic review. The search process and exclusion reasons are described in **Figure 1**.

The included articles were published between 2013 and 2020. The quality assessment tools used in these articles included the Cochrane Risk of Bias tool, GRADE, and Jadad. The basic details of the included studies are presented in **Table 1**.

**Table 1:** Basic information of the included meta-analyses.

Reference	Meta-analysis examined	Antipsychotic causing hyperprolactinemia	Population	Intervention		Outcomes	Quality assessment tool	Introduction
				Experimental	Control			
1. Li, X. 2013 (27)	5 RCTs	Haloperidol Sulpiride Risperidone Quetiapine	Adult patients with antipsychotic-induced hyperprolactinemia	Aripiprazole	Placebo	Adverse events, efficacy of treatment	GRADE	Five RCTs were included to compare the safety and efficacy of aripiprazole vs. placebo in the adjunctive treatment of antipsychotic-induced hyperprolactinemia. Results: Adjunctive aripiprazole demonstrated a substantial 79.11% rate of prolactin level normalization. Meta-analysis indicated comparable side effect profiles between the adjunctive aripiprazole and placebo groups, except for higher occurrences of sedation, insomnia, and headache observed with adjunctive aripiprazole doses exceeding 15 mg/day. Main conclusion: Adding aripiprazole is a safe and effective way to reduce prolactin level. The most appropriate dose of aripiprazole may be 5 mg/day.
2. Meng, M. 2015 (28)	21 RCTs	Haloperidol Sulpiride Perphenazine Chlorpromazine Risperidone Quetiapine Clozapine Olanzapine Paliperidone	Patients with schizophrenia and confirmed to have antipsychotic-induced hyperprolactinemia through blood tests	Aripiprazole	Placebo or APs monotherapy	HPL recovery	Cochrane Risk of Bias tool GRADE	Updated meta-analysis conducted to assess the safety and efficiency of adjunctive aripiprazole for antipsychotic-induced hyperprolactinemia. Results: Adjunctive aripiprazole significantly increased the proportion of patients with hyperprolactinemia recovery compared to the control condition. While the adverse effect rates during follow-up were similar between groups, the aripiprazole group exhibited higher likelihoods of reporting somnolence and headaches. High-dose aripiprazole (>5mg/day) appeared more effective than low-dose (<5mg/day), though the difference was not statistically significant. Main conclusion: Adding aripiprazole is both safe and effective for treating antipsychotic-induced hyperprolactinemia.
3. Bo, Q. J. 2016 (29)	2 Observational studies 1 RCT	Sulpiride Chlorpromazine Clozapine Olanzapine Risperidone Quetiapine	Adult patients with antipsychotic-induced hyperprolactinemia	Metformin	Placebo	Serum prolactin level, prolactin-related side effects	Cochrane Risk of Bias tool	Systematic review evaluating the efficacy and safety of adding metformin to treat antipsychotic-induced hyperprolactinemia. A search yielded three trials involving 325 patients, including one randomized clinical trial and two observational studies. Because only three studies were included, meta-analysis could not be performed. Results: Adjunctive metformin exhibited a notable reduction in serum prolactin levels. The RCT showed a 67% resumption of menstruation in those with menstrual disturbances compared to 5% in the placebo group. Observational data indicated that 91% of patients no longer exhibited signs or symptoms of galactorrhea. Adverse drug reactions in the RCT were similar between metformin and placebo, except for non-discontinuation-associated increases in nausea, insomnia, and agitation. Main conclusion: Adjunctive metformin can significantly reduce prolactin levels and alleviate prolactin-related symptoms.
4. Zheng, W. 2017 (30)	4 RCTs	Sulpiride Risperidone Clozapine Olanzapine Quetiapine	Adult patients with schizophrenia diagnosed any diagnostic criteria	Metformin	Placebo or APs monotherapy	Serum prolactin level, prolactin-related symptoms	Cochrane Risk of Bias tool Jadad GRADE	Meta-analysis evaluating adjunctive metformin in treatment of AP-associated hyperprolactinemia. Results: The metformin group exhibited a significantly lower serum prolactin level at the endpoint. The rate of menstruation resumption in patients with menstrual disturbances was notably higher at 66.7% in the metformin group compared to 4.8% in the control group. Adverse drug reactions and all-cause discontinuation were similar between the metformin and control groups. Main conclusion: Adjunctive metformin is effective and safe for reducing hyperprolactinemia and prolactin-related symptoms.
5. Labad, J. 2020 (31)	7 RCTs 18 Open-label studies 1 Study with one open-label arm and two randomized arms	Haloperidol Risperidone Sulpiride Amisulpride Other multiple antipsychotics	Patients with psychotic disorders who had hyperprolactinemia	1. Switch to antipsychotics that have less effect on prolactin 2. Aripiprazole 3. dopamine agonists 4. metformin	Placebo or maintaining antipsychotic treatment	Prolactin concentrations	Cochrane Risk of Bias tool	Comparison of four different strategies for lowering prolactin concentration. Meta-analyses were performed only on RCTs that maintained the original antipsychotics or used placebo for comparison groups. Results: Five randomized controlled trials investigating the addition of aripiprazole showed a substantial reduction in prolactin concentration compared to both placebo and the maintenance of antipsychotic treatment. Additionally, the analysis of three placebo-controlled RCTs indicated comparable withdrawal rates for aripiprazole (10.1%) and placebo (11.5%), emphasizing that, according to the available evidence, the primary consideration for reducing prolactin concentrations in schizophrenia patients with hyperprolactinemia is the addition of aripiprazole. Main conclusion: Adding aripiprazole is the preferred option for reducing prolactin concentration in patients with hyperprolactinemia.

RCT, randomized controlled trial; AP, antipsychotic; GRADE, Grading of Recommendations, Assessment, Development and Evaluation.

### 3.2 Search strategy assessment

The electronic bibliographic databases utilized in the five included meta-analyses included Pubmed, Embase, Cochrane, CKNI, Wanfang database, and others. The details of the search methodology applied in each meta-analyses are summarized in **Table 2**.

**Table 2:** Databases searched by each of the included meta-analyses.

	PubMed	Embase	SCI	Cochrane	CKNI	Scopus	Clinical Trials.gov	Others
1. Li, X. 2013 (27)	√	√		√	√			CBM
2. Meng, M. 2015 (28)	√	√		√	√		√	EBSCO, Chongqing VIP, Wangfang, CBM, Taiwan Electronic Periodical Services
3. Bo, Q. J. 2016 (29)	√	√	√	√	√		√	Wanfang, CBM, WHO ICTRP, Chinese Clinical Trials Register.
4. Zheng, W. 2017 (30)	√	√		√	√			PsycINFO, CMB, Wanfang
5. Labad, J. 2020 (31)	√					√	√	PsycINFO

SCI, Science citation index; CKNI, China National Knowledge Infrastructure; CBM, Chinese Biomedical Literature; EBSCO, EBSCOhost; WHO ICTRP, World Health Organization International Clinical Trials Registry Platform.

### 3.3 PRISMA quality of the included meta-analyses

The PRISMA scores for the included meta-analyses ranged from 19.5–26 (average score, 22.4). All included meta-analyses had scores higher than 15, indicating there were no serious defects. Two meta-analyses (29, 30) had scores in the range of 15 to <21, which reflects some defects. The other three meta-analyses (27, 28, 31) were relatively more complete and had scores higher than 21. The details of the PRISMA quality scores of the included meta-analyses are presented in **Table 3**.

**Table 3:** PRISMA quality scores of included meta-analyses.

	Li, X. 2013 (27)	Meng, M. 2015 (28)	Bo, Q. J. 2016 (29)	Zheng, W. 2017 (30)	Labad, J. 2020 (31)
**1. Title**	1	1	1	1	1
**2. Structured abstract**	1	1	0.5	0.5	0.5
**3. Rational**	1	1	1	1	1
**4. Objectives**	1	1	1	1	1
**5. Protocol and registration**	0	0	1	0	1
**6. Eligibility criteria**	0.5	1	0.5	1	1
**7. Information sources**	1	1	1	1	1
**8. Search**	0.5	0.5	0.5	0.5	1
**9. Study selection**	1	1	1	0.5	1
**10. Data collection process**	1	1	1	0.5	1
**11. Data items**	0.5	1	1	0	1
**12. Risk of bias in individual studies**	1	1	1	1	1
**13. Summary measures**	0	1	0	1	1
**14. Synthesis of results**	1	1	0	1	1
**15. Risk of bias across studies**	1	1	1	1	1
**16. Additional analyses**	1	1	0	0	1
**17. Study selection**	1	1	1	1	1
**18. Study characteristics**	1	1	1	1	1
**19. Risk of bias within studies**	1	1	1	1	1
**20. Results of individual studies**	1	1	1	1	1
**21. Synthesis of results**	1	1	0	1	1
**22. Risk of bias across studies**	0	1	0	0	0.5
**23. Additional analysis**	1	1	0	0	1
**24. Summary of evidence**	0.5	0.5	1	0.5	1
**25. Limitations**	1	1	1	1	1
**26. Conclusions**	1	1	1	1	1
**27. Funding**	1	1	1	1	1
**Total**	22	25	19.5	19.5	26

### 3.4 AMSTAR 2 assessment of the included meta-analyses

The AMSTAR 2 quality evaluation of the five included meta-analyses is presented in **Table 4**. Two meta-analyses (29, 31) were of low quality, and three (27, 28, 30) were of very low quality. The main reason for the low quality was that none of the meta-analyses provided a list of excluded articles and explained the reasons (item 7). Other reasons for the reduced quality were that three meta-analyses (27, 28, 30) did not register and publish their research protocol in advance (item 2) and two meta-analyses (27, 30) failed to assess the publication bias because of the limited number of RCTs (item 15).

**Table 4:** AMSTAR 2 quality evaluation of included meta-analyses.

	Li, X. 2013 (27)	Meng, M. 2015 (28)	Bo, Q. J. 2016 (29)	Zheng, W. 2017 (30)	Labad, J. 2020 (31)
**1. PICO**	Y	Y	Y	Y	Y
**2. Explicit statement**	N	N	Y	N	Y
**3. Selection of the study designs**	N	N	N	N	N
**4. Search study**	PY	Y	PY	PY	PY
**5. Study selection in duplicate**	Y	Y	Y	Y	Y
**6. Data extraction in duplicate**	Y	Y	Y	Y	Y
**7. List of excluded studies**	N	N	N	N	N
**8. Description of included studies**	Y	Y	Y	PY	Y
**9. Risk of bias in individual studies**	Y	Y	Y	Y	Y
**10. Funding of individual studies**	N	N	N	N	N
**11. Methods for statistical combination**	Y	Y	NMC	Y	Y
**12. Assessment of potential impact of risk of bias in individual studies**	Y	Y	NMC	N	Y
**13. Account for risk of bias when interpreting the result**	Y	Y	Y	Y	Y
**14. Explanation for heterogeneity**	Y	Y	Y	Y	Y
**15. Publication bias**	N	Y	NMC	N	Y
**16. Conflict of interest**	Y	Y	Y	Y	Y
**Credibility**	VL	VL	L	VL	L

Y, Yes; N, No; PY, Partial yes; NMC, No meta-analysis conducted; L, Low; VL, Very low; PICO, Population, Intervention, Control group, Outcome.

### 3.5 Clinical evidence for treatment of antipsychotic-induced hyperprolactinemia

The five meta-analyses included in this review suggested that adjunctive aripiprazole, dopamine agonists, and metformin can effectively reduce serum prolactin concentrations. Among the included meta-analyses, Labad et al. (31) had the highest quality scores, indicating this meta-analysis was of the highest clinical significance. Li et al. (27) and Meng et al. (28) had rather high PRISMA scores, indicating that they have certain value for clinical guidance. Due to the insufficient studies included, meta-analysis was not conducted in Bo et al. (29), which resulted in decreased quality scores. Zheng et al., 2017 (30) exhibited several article structural defects, resulting in lower scores during evaluation. The PRISMA scores for the included meta-analyses suggested that all the reports were relatively comprehensive and accurate. However, the AMSTAR 2 scores were generally low, indicating methodological deficiencies in the reports. These shortcomings encompassed a lack of an exclusion list, a failure to analyze publication bias, and so on. It implied that the included meta-analyses demonstrated a relatively objective process of secondary analysis. However, the methodological flaws contributed to a certain degree of reduction in the credibility of the conclusions.

#### 3.5.1 Adjunct aripiprazole

Three of the meta-analyses (27, 28, 31) discussed the efficacy of adjunctive aripiprazole and came to a positive conclusion that aripiprazole can effectively reduce the serum prolactin level in patients with antipsychotic-induced hyperprolactinemia. All of the three meta-analyses also assessed the safety of adjunctive aripiprazole and reached a similar conclusion that adjunctive aripiprazole was generally safe and well tolerated. While the PRISMA scores for these three meta-analyses were high, ranging from 22 to 26, the AMSTAR 2 quality varied, with one meta-analysis rated low and the other two very low. This suggests a need for further enhancement in the methodology of each meta-analysis.

Li et al. (27) demonstrated a significant 79.11% rate of prolactin level normalization with adjunctive aripiprazole. Meta-analysis indicated comparable side effect profiles between the adjunctive aripiprazole and placebo groups, except for higher occurrences of sedation, insomnia, and headache observed with adjunctive aripiprazole doses exceeding 15 mg/day. Furthermore, despite aripiprazole’s partial agonist activity at D2 receptors, adjunctive treatment did not lead to clinical deterioration or symptom exacerbation in hyperprolactinemia cases in this meta-analysis. The findings affirmed the safety and good tolerability of adjunctive aripiprazole (5–10 mg/day) in addressing antipsychotic-induced hyperprolactinemia. Notably, the meta-analysis recommended vigilant monitoring for side effects such as sedation, insomnia, and headache during adjunctive aripiprazole treatment. Li et al. (27) further explored the most appropriate dose of adjunctive aripiprazole and found that it may be 5 mg/day.

Meng et al. (28) reported comparable findings. They suggested that adjunctive aripiprazole could significantly increase the proportion of patients whose prolactin level returned to the normal range compared to the control condition. While the adverse effect rates during follow-up were similar between groups, the aripiprazole group exhibited higher likelihoods of reporting somnolence and headaches. In the investigation of the ideal dosage, high-dose aripiprazole (>5mg/day) appeared more effective than low-dose (<5mg/day) in promoting recovery from hyperprolactinemia, though the difference was not statistically significant. The findings provided reassurance that the use of adjunctive aripiprazole did not exacerbate existing psychotic symptoms.

Labad et al. (31) claimed that of all the potential therapeutic strategies for lowering prolactin, clinical trials prominently addressed the addition of aripiprazole to antipsychotic treatment, positioning it as the first option based on evidence-based medicine levels. The safety profile of aripiprazole was well-explored, revealing no significant differences compared to placebo. This meta-analysis further demonstrated low rates of psychopathological worsening with the open-label studies suggested a withdrawal rate of approximately 5% attributed to psychopathology worsening. Additionally, the analysis of three placebo-controlled RCTs indicated comparable withdrawal rates for aripiprazole (10.1%) and placebo (11.5%), emphasizing that, according to the available evidence, the primary consideration for reducing prolactin concentrations in schizophrenia patients with hyperprolactinemia is the addition of aripiprazole. In this meta-analysis, the doses of adjunctive aripiprazole ranged from 5 to 30 mg/day. However, this meta-analysis did not extensively investigate the optimal dosage of aripiprazole. Only one study included in the meta-analysis suggests that the effect size of aripiprazole dosage of 10 or 20 mg/day is greater compared to 5 mg/day.

#### 3.5.2 Adjunct metformin

Two of the included meta-analyses (29, 30) evaluated the use of metformin in the treatment of hyperprolactinemia. The PRISMA scores for both meta-analyses were 19.5, and the AMSTAR 2 quality evaluations were low quality and very low quality, respectively. Both meta-analyses suggested that adjunctive metformin appeared to be effective and safe for reducing prolactin and improving prolactin-related symptoms. The meta-analysis by Bo et al. (29) included three clinical studies, with metformin doses of 750, 1000, and 1500 mg/day respectively. They suggested that adjunctive metformin exhibited a notable reduction in serum prolactin levels, averaging 54.6 μg/L in the three trials. The RCT showed a 67% resumption of menstruation in those with menstrual disturbances compared to 5% in the placebo group. Observational data indicated that 91% of patients no longer exhibited signs or symptoms of galactorrhea. Adverse drug reactions in the RCT were similar between metformin and placebo, except for non-discontinuation-associated increases in nausea, insomnia, and agitation.

In the meta-analysis conducted by Zheng et al. (30), the average dosage of metformin was 1167 mg/day (ranging from 750 to 1500 mg/day), and the results showed an average decrease in prolactin at endpoint of 6.87 μg/L. They also observed a menstruation resumption rate of 66.7% among patients with menstrual disturbances, compared to 4.8% in the control group. The incidence of adverse drug reactions and the overall discontinuation rate were similar between the metformin and control groups.

#### 3.5.3 Adjunct dopamine agonists

Among all included meta-analyses, only Labad et al. (31) assessed the effectiveness and safety of dopamine agonists. They investigated Cabergoline (dose range 0.125mg/week-1mg/day), Bromocriptine (dose range 5-40mg/day), and Terguride (dose range 1mg/day), finding that all three dopamine agonists could lower serum prolactin levels. Among them, Cabergoline had the most substantial clinical evidence for reducing serum prolactin, while Terguride had slightly lower effect sizes for prolactin reduction. Regarding safety, dopamine agonists exhibited safety profiles exceeding expectations. The overall withdrawal rate for Cabergoline was 2.9% (with no psychotic relapse), for Bromocriptine was 20% (with psychopathology worsening at 13.3%), and for Terguride was 13% (all due to psychopathology worsening).

## 4 Discussion

Systematic review is an important research method to determine the best sources of evidence. However, only high-quality systematic reviews can provide scientific evidence for use by healthcare providers. The objective of the present review was to conduct an umbrella review of overlapping meta-analyses and systematic reviews of treatments for antipsychotic-induced hyperprolactinemia to determine which article(s) provide the best available evidence for selecting treatments. To date, the Food and Drug Administration (FDA) has not approved any therapeutic strategy for the treatment of antipsychotic-induced hyperprolactinemia. Therefore, it is very important to summarize the findings and quality of previous clinical studies, in order to help clinicians choose the most suitable treatment for their patients. From our literature searches, five articles were included in this umbrella review, and these meta-analyses reported that aripiprazole, metformin and dopamine agonists may be effective at reducing prolactin concentrations in patients with antipsychotic-induced hyperprolactinemia.

Aripiprazole as a dopamine receptor stabilizer is widely regarded as having the ability to lower serum prolactin levels. In a meta-analysis of 32 RCTs examining various antipsychotics’ impact on prolactin levels in children and adolescents, it was found that only aripiprazole significantly decreased serum prolactin levels (32). Moreover, aripiprazole is supported by the most extensive clinical evidence for treating antipsychotic-induced hyperprolactinemia. The Chinese Society of Neuroscience & Psychiatry, Schizophrenia Clinical Research Alliance released a consensus on the management of antipsychotic-induced hyperprolactinemia in 2021 (20). In the consensus, adjunctive aripiprazole was hailed as the most effective intervention among all therapeutic measures. This conclusion aligns with the findings from this umbrella review. All the clinical evidence included in this review once again validates the safety and efficacy of adjunctive aripiprazole for the treatment of antipsychotic-induced hyperprolactinemia. Currently, there is no conclusive evidence for the optimal dosage of aripiprazole in treating hyperprolactinemia, but most studies suggest that low-dose aripiprazole have advantages over higher doses. This may be related to the fact that low-dose aripiprazole have already occupied most D2 receptors in the striatum (33). More large-sample clinical studies are needed to further explore the dose-response relationship of aripiprazole in treating hyperprolactinemia. It is noteworthy that the decrease in serum prolactin abnormalities induced by aripiprazole may serve as a biomarker for the rebound of positive symptoms in patients with schizophrenia. A clinical study suggests that after switching to aripiprazole treatment, patients with abnormally low prolactin levels experience a significantly higher rebound rate of psychotic symptoms compared to patients without abnormally low prolactin levels (34). Therefore, monitoring serum prolactin levels during treatment may help predict later rebound of psychotic symptoms.

As a first-line drug for diabetes, the effects of metformin for improving antipsychotic-induced weight gain and abnormal glucose and lipid metabolism had been demonstrated by many studies (35). However, its effect on hyperprolactinemia has not been clearly established. One study showed that metformin can improve the endogenous dopaminergic tone in female patients with polycystic ovary syndrome (36). Building on this potential mechanism, developing studies have focused on whether metformin can reduce prolactin levels. The limited clinical evidence provided by the two meta-analyses included in our review (29, 30) suggests that metformin shows promise as a treatment for antipsychotic-induced hyperprolactinemia. Although the current evidence for the adjunctive metformin in the treatment of hyperprolactinemia is insufficient, there have been an increasing number of clinical trials exploring its effectiveness and safety as a potential drug. Recently, a randomized controlled trial assessed the efficacy of metformin in treating hyperprolactinemia induced by amisulpride, yielding positive conclusions that metformin can effectively reduce serum prolactin levels without significant adverse effects (37). This study once again provided compelling clinical evidence for the efficacy of metformin in reducing prolactin. Additionally, clinical evidence suggests that metformin, while improving antipsychotic-induced metabolic syndrome in patients with schizophrenia, also has a role in improving psychiatric and cognitive symptoms (38). This indicates that the benefits of metformin for patients may be multidimensional and worth exploring further. In the future, more research should focus on the potential of metformin, thereby expanding the possibilities for treating antipsychotic-induced hyperprolactinemia.

Dopamine agonists have been regarded as an important treatment of hyperprolactinemia since the invention of bromocriptine (39). In addition to drug-induced hyperprolactinemia, dopamine agonists have established indications for treatment of physiological hyperprolactinemia and hyperprolactinemia caused by pituitary prolactin adenoma and other reasons. However, the mechanisms of action of dopamine agonists and antipsychotics are conflicting to a certain extent. Dopamine agonists were reported to potentially aggravate schizophrenia (40, 41). For this reason, when considering adjunctive dopamine receptor agonists for the treatment of hyperprolactinemia, special attention should be paid to the drug’s safety profile. In the present umbrella review, Labad et al. (31) assessed the safety and efficacy of adding dopamine agonists and reported psychopathological worsening rates of 13.3% for bromocriptine and 13% for terguride, but no psychotic relapses in patients treated with cabergoline. While these rates of psychopathological worsening were lower than expected, this outcome was consistent with an earlier study exploring the safety of dopamine agonists (42), in which only 8 cases (1.3%) experienced psychotic side effects in a sample of 600 patients using dopamine agonists. Another study reviewed four pediatric cases of risperidone-induced hyperprolactinemia treated with cabergoline and found that cabergoline was well tolerated (43). These studies suggest that dopamine receptor agonists are generally safe for psychiatric patients.

Presently, in the treatment of antipsychotic-induced hyperprolactinemia, existing clinical studies are evolving in two directions. Firstly, by substantiating reliable treatment methods with larger sample sizes, such as adjunctive aripiprazole, or by conducting more in-depth subgroup analyses to explore optimal treatment dosages. Secondly, by attempting to introduce more treatment methods to explore diverse treatment modalities. This suggests that antipsychotic-induced hyperprolactinemia is increasingly drawing attention from clinicians, and investigating its diverse and standardized treatment methods will hold significant clinical value. With the progress of studies, some novel treatment approaches are also gaining increased attention, such as adding the Peony-Glycyrrhiza decoction (PGD) (44), adjunctive high-dose vitamin B6 (45), and so on.

PGD is a traditional Chinese medicine formulated with peony and glycyrrhiza. It is believed to have the effect of reversing the decrease in estradiol levels caused by prolactin (46). A prior network meta-analysis assessed the efficacy of PGD in reducing prolactin levels, suggesting that while PGD may not be as effective as aripiprazole, it still has a significant effect in lowering prolactin levels. Furthermore, in subgroup analysis, PGD demonstrated more notable effects than other treatments in patients with risperidone-induced hyperprolactinemia (47). The findings confirmed the effectiveness of PGD treatment while also introducing a new concept that there may be different underlying mechanisms for hyperprolactinemia induced by different antipsychotics, thus requiring consideration of diverse treatments.

Vitamin B6 plays a crucial role in cellular metabolism and stress response. Clinical research has now begun to explore its clinical value in treating hyperprolactinemia (45). A recent network meta-analysis comprehensively evaluated all treatment measures for hyperprolactinemia, affirming the effectiveness of traditional methods such as aripiprazole while also proposing the potential of high-dose B6 treatment for hyperprolactinemia. Moreover, the study indicated that different treatments have varying efficacy for patients with different prolactin levels, and patients with initial prolactin levels below 50 ng/ml may not require specific interventions (48). This suggests that in the future, a more precise treatment model for hyperprolactinemia should consider the initial prolactin levels of patients.

## 5 Limitations

This umbrella review has some limitations. Firstly, we only included meta-analyses and systematic reviews, and thus, it was not possible to examine outcomes at the patient level. Secondly, due to the low number of RCTs included in some articles, meta-analysis could not be conducted, thus reducing the quality. Thirdly, some articles included and analyzed low-quality RCTs, which may affect the validity of the conclusions.

## 6 Conclusion

Adding aripiprazole or a dopamine agonist can effectively and safely improve antipsychotic-induced hyperprolactinemia. There is clinical evidence indicating that adjunctive metformin can also reduce the serum prolactin concentration, but more clinical trials are needed to confirm this finding. Adjunctive dopamine agonists have been proven to be effective and safe for the treatment of antipsychotic-induced hyperprolactinemia. Among those evaluated, aripiprazole may be the most appropriate.

## Author contributions

QJ: Writing – original draft. TL: Data curation, Writing – review & editing. LZ: Methodology, Supervision, Writing – review & editing. YS: Data curation, Writing – review & editing. ZM: Investigation, Methodology, Writing – review & editing. YX: Investigation, Data curation, Writing – review & editing. CW: Supervision, Writing – review & editing. QB: Conceptualization, Supervision, Writing – review & editing.

## References

[1] Jauhar S Johnstone M McKenna PJ. Schizophrenia. Lancet (London England). (2022) 399:473–86. doi: 10.1016/S0140-6736(21)01730-X 35093231

[2] Lisoway AJ Chen CC Zai CC Tiwari AK Kennedy JL. Toward personalized medicine in schizophrenia: Genetics and epigenetics of antipsychotic treatment. Schizophr Res. (2021) 232:112–24. doi: 10.1016/j.schres.2021.05.010 34049235

[3] Lieberman JA Bymaster FP Meltzer HY Deutch AY Duncan GE Marx CE. Antipsychotic drugs: comparison in animal models of efficacy, neurotransmitter regulation, and neuroprotection. Pharmacol Rev. (2008) 60:358–403. doi: 10.1124/pr.107.00107 18922967 PMC4821196

[4] Missale C Nash SR Robinson SW Jaber M Caron MG. Dopamine receptors: from structure to function. Physiol Rev. (1998) 78:189–225. doi: 10.1152/physrev.1998.78.1.189 9457173

[5] Beaulieu JM Gainetdinov RR. The physiology, signaling, and pharmacology of dopamine receptors. Pharmacol Rev. (2011) 63:182–217. doi: 10.1124/pr.110.002642 21303898

[6] Meltzer HY. New trends in the treatment of schizophrenia. CNS Neurol Disord Drug Targets. (2017) 16:900–6. doi: 10.2174/1871527316666170728165355 28758583

[7] Halbreich U Kinon BJ Gilmore JA Kahn LS. Elevated prolactin levels in patients with schizophrenia: mechanisms and related adverse effects. Psychoneuroendocrinology. (2003) 28 Suppl 1:53–67. doi: 10.1016/s0306-4530(02)00112-9 12504072

[8] Society TCN. [Consensus on diagnosis and treatment of hyperprolactin]. Natl Med J China. (2011) 91:147–54. doi: 10.3760/cma.j.issn.0376-2491.2011.03.002

[9] Peveler RC Branford D Citrome L Fitzgerald P Harvey PW Holt RI. Antipsychotics and hyperprolactinaemia: clinical recommendations. J Psychopharmacol (Oxford England). (2008) 22:98–103. doi: 10.1177/0269881107087346 18477626

[10] Halbreich U Kahn LS. Hyperprolactinemia and schizophrenia: mechanisms and clinical aspects. J Psychiatr Practice. (2003) 9:344–53. doi: 10.1097/00131746-200309000-00003 15985953

[11] Montejo ÁL Arango C Bernardo M Carrasco JL Crespo-Facorro B Cruz JJ. Multidisciplinary consensus on the therapeutic recommendations for iatrogenic hyperprolactinemia secondary to antipsychotics. Front Neuroendocrinol. (2017) 45:25–34. doi: 10.1016/j.yfrne.2017.02.003 28235557

[12] Holt RI Peveler RC. Antipsychotics and hyperprolactinaemia: mechanisms, consequences and management. Clin Endocrinol. (2011) 74:141–7. doi: 10.1111/j.1365-2265.2010.03814.x 20455888

[13] Kinon BJ Gilmore JA Liu H Halbreich UM. Prevalence of hyperprolactinemia in schizophrenic patients treated with conventional antipsychotic medications or risperidone. Psychoneuroendocrinology. (2003) 28 Suppl 2:55–68. doi: 10.1016/s0306-4530(02)00127-0 12650681

[14] Montgomery J Winterbottom E Jessani M Kohegyi E Fulmer J Seamonds B. Prevalence of hyperprolactinemia in schizophrenia: association with typical and atypical antipsychotic treatment. J Clin Psychiatry. (2004) 65:1491–8. doi: 10.4088/jcp.v65n1108 15554761

[15] Seeman P. Atypical antipsychotics: mechanism of action. Can J Psychiatry Rev Can Psychiatrie. (2002) 47:27–38. doi: 10.1177/070674370204700106 11873706

[16] Kapur S Langlois X Vinken P Megens AA De Coster R Andrews JS. The differential effects of atypical antipsychotics on prolactin elevation are explained by their differential blood-brain disposition: a pharmacological analysis in rats. J Pharmacol Exp Ther. (2002) 302:1129–34. doi: 10.1124/jpet.102.035303 12183672

[17] Redman B Kitchen C Johnson KW Bezwada P Kelly DL. Levels of prolactin and testosterone and associated sexual dysfunction and breast abnormalities in men with schizophrenia treated with antipsychotic medications. J Psychiatr Res. (2021) 143:50–3. doi: 10.1016/j.jpsychires.2021.08.022 34450525

[18] Zhang C Mao Y Song L. Precise treatments for schizophrenia: where is the way forward? Gen Psychiatry. (2018) 31:e000002. doi: 10.1136/gpsych-2018-000002 PMC6211279 30582112

[19] Rusgis MM Alabbasi AY Nelson LA. Guidance on the treatment of antipsychotic-induced hyperprolactinemia when switching the antipsychotic is not an option. Am J Health-system Pharmacy: AJHP: Off J Am Soc Health-System Pharmacists. (2021) 78:862–71. doi: 10.1093/ajhp/zxab065 PMC7989660 33954421

[20] Chinese Society of Neuroscience & Psychiatry SCRA. Consensus on the management of antipsychotic−induced hyperprolactinemia. Chin J Psychiatry. (2021) 54:163–9. doi: 10.3760/cma.j.cn113661-20201219-00514

[21] Croxtall JD. Aripiprazole: a review of its use in the management of schizophrenia in adults. CNS Drugs. (2012) 26:155–83. doi: 10.2165/11208400-000000000-00000 22296317

[22] Zhang CH Ma K Yuan BC Yuan Y Chen YX. Bushen Huoxue herbal medicine for treating hyperprolactinemia in women: a Meta-analysis. China J Chin Mater Med. (2019) 44:1087–93. doi: 10.19540/j.cnki.cjcmm.20190125.001 30989968

[23] Wei Y La L Wang L Batey R Wang C Li Y. Paeoniflorin and liquiritin, two major constituents in Chinese herbal formulas used to treat hyperprolactinemia-associated disorders, inhibits prolactin secretion in prolactinoma cells by different mechanisms. J Ethnopharmacol. (2017) 204:36–44. doi: 10.1016/j.jep.2017.03.054 28396166

[24] Huang X Ren L Hou L Fan H Wang C Wang C. Paeoniflorin ameliorates antipsychotic-induced hyperprolactinemia in rats by attenuating impairment of the dopamine D2 receptor and TGF-β1 signaling pathways in the hypothalamus and pituitary. J Ethnopharmacol. (2020) 257:112862. doi: 10.1016/j.jep.2020.112862 32294507

[25] Moher D Liberati A Tetzlaff J Altman DG. Preferred reporting items for systematic reviews and meta-analyses: the PRISMA statement. BMJ (Clin Res ed). (2009) 339:b2535. doi: 10.1136/bmj.b2535 PMC2714657 19622551

[26] Shea BJ Grimshaw JM Wells GA Boers M Andersson N Hamel C. Development of AMSTAR: a measurement tool to assess the methodological quality of systematic reviews. BMC Med Res Methodol. (2007) 7:10. doi: 10.1186/1471-2288-7-10 17302989 PMC1810543

[27] Li X Tang Y Wang C. Adjunctive aripiprazole versus placebo for antipsychotic-induced hyperprolactinemia: meta-analysis of randomized controlled trials. PloS One. (2013) 8:e70179. doi: 10.1371/journal.pone.0070179 23936389 PMC3731351

[28] Meng M Li W Zhang S Wang H Sheng J Wang J. Using aripiprazole to reduce antipsychotic-induced hyperprolactinemia: meta-analysis of currently available randomized controlled trials. Shanghai Arch Psychiatry. (2015) 27:4–17. doi: 10.11919/j.issn.1002-0829.215014 25852251 PMC4372756

[29] Bo QJ Wang ZM Li XB Ma X Wang CY de Leon J. Adjunctive metformin for antipsychotic-induced hyperprolactinemia: A systematic review. Psychiatry Res. (2016) 237:257–63. doi: 10.1016/j.psychres.2016.01.031 26822064

[30] Zheng W Yang XH Cai DB Ungvari GS Ng CH Wang N. Adjunctive metformin for antipsychotic-related hyperprolactinemia: A meta-analysis of randomized controlled trials. J Psychopharmacol (Oxford England). (2017) 31:625–31. doi: 10.1177/0269881117699630 28372526

[31] Labad J Montalvo I Gonzalez-Rodriguez A Garcia-Rizo C Crespo-Facorro B Monreal JA. Pharmacological treatment strategies for lowering prolactin in people with a psychotic disorder and hyperprolactinaemia: A systematic review and meta-analysis. Schizophr Res. (2020) 222:88–96. doi: 10.1016/j.dib.2020.105904 32507371

[32] Krøigaard SM Clemmensen L Tarp S Pagsberg AK. A meta-analysis of antipsychotic-induced hypo- and hyperprolactinemia in children and adolescents. J Child Adolesc Psychopharmacol. (2022) 32:374–89. doi: 10.1089/cap.2021.0140 36074098

[33] Gründer G Fellows C Janouschek H Veselinovic T Boy C Bröcheler A. Brain and plasma pharmacokinetics of aripiprazole in patients with schizophrenia: an [18F]fallypride PET study. Am J Psychiatry. (2008) 165:988–95. doi: 10.1176/appi.ajp.2008.07101574 18381901

[34] Jen YW Hwang TJ Chan HY Hsieh MH Liu CC Liu CM. Abnormally low prolactin levels in schizophrenia patients after switching to aripiprazole in a randomized trial: a biomarker for rebound in psychotic symptoms? BMC Psychiatry. (2020) 20:552. doi: 10.1186/s12888-020-02957-7 33228575 PMC7686669

[35] Bushe CJ Bradley AJ Doshi S Karagianis J. Changes in weight and metabolic parameters during treatment with antipsychotics and metformin: do the data inform as to potential guideline development? A systematic review of clinical studies. Int J Clin Practice. (2009) 63:1743–61. doi: 10.1111/ijcp.2009.63.issue-12 19840151

[36] Ortega-González C Cardoza L Coutiño B Hidalgo R Arteaga-Troncoso G Parra A. Insulin sensitizing drugs increase the endogenous dopaminergic tone in obese insulin-resistant women with polycystic ovary syndrome. J Endocrinol. (2005) 184:233–9. doi: 10.1677/joe.1.05844 15642799

[37] Zhu C Li R Ju M Xiao X Yuan TF Jin Z. Metformin in the treatment of amisulpride-induced hyperprolactinemia: A clinical trial. Front Mol Neurosci. (2022) 15:892477. doi: 10.3389/fnmol.2022.892477 35721320 PMC9205636

[38] Battini V Cirnigliaro G Leuzzi R Rissotto E Mosini G Benatti B. The potential effect of metformin on cognitive and other symptom dimensions in patients with schizophrenia and antipsychotic-induced weight gain: a systematic review, meta-analysis, and meta-regression. Front Psychiatry. (2023) 14:1215807. doi: 10.3389/fpsyt.2023.1215807 37502816 PMC10370497

[39] Dekkers OM Lagro J Burman P Jørgensen JO Romijn JA Pereira AM. Recurrence of hyperprolactinemia after withdrawal of dopamine agonists: systematic review and meta-analysis. J Clin Endocrinol Metab. (2010) 95:43–51. doi: 10.1210/jc.2009-1238 19880787

[40] Dorevitch A Aronzon R Stark M. Psychotic exacerbation attributed to low-dose bromocriptine treatment of galactorrhea and hyperprolactinemia. Acta Obstetricia Gynecol Scandinavica. (1991) 70:375–6. doi: 10.3109/00016349109007893 1746267

[41] Snellen M Power J Blankley G Galbally M. Pharmacological lactation suppression with D2 receptor agonists and risk of postpartum psychosis: A systematic review. Aust New Z J Obstetrics Gynaecol. (2016) 56:336–40. doi: 10.1111/ajo.12479 27297803

[42] Turner TH Cookson JC Wass JA Drury PL Price PA Besser GM. Psychotic reactions during treatment of pituitary tumours with dopamine agonists. Br Med J (Clin Res ed). (1984) 289:1101–3. doi: 10.1136/bmj.289.6452.1101 PMC1443261 6435792

[43] Cohen LG Biederman J. Treatment of risperidone-induced hyperprolactinemia with a dopamine agonist in children. J Child Adolesc Psychopharmacol. (2001) 11:435–40. doi: 10.1089/104454601317261618 11838826

[44] Yang P Li L Yang D Wang C Peng H Huang H. Effect of peony-glycyrrhiza decoction on amisulpride-induced hyperprolactinemia in women with schizophrenia: A preliminary study. Evidence-Based Complementary Altern Med: eCAM. (2017) 2017:7901670. doi: 10.1155/2017/7901670 PMC5727636 29317896

[45] Zhuo C Xu Y Wang H Fang T Chen J Zhou C. Safety and efficacy of high-dose vitamin B6 as an adjunctive treatment for antipsychotic-induced hyperprolactinemia in male patients with treatment-resistant schizophrenia. Front Psychiatry. (2021) 12:681418. doi: 10.3389/fpsyt.2021.681418 34512411 PMC8426548

[46] Yamada K Kanba S Yagi G Asai M. Effectiveness of herbal medicine (shakuyaku-kanzo-to) for neuroleptic-induced hyperprolactinemia. J Clin Psychopharmacol. (1997) 17:234–5. doi: 10.1097/00004714-199706000-00025 9169978

[47] Zhang L Qi H Xie YY Zheng W Liu XH Cai DB. Efficacy and safety of adjunctive aripiprazole, metformin, and paeoniae-glycyrrhiza decoction for antipsychotic-induced hyperprolactinemia: A network meta-analysis of randomized controlled trials. Front Psychiatry. (2021) 12:728204. doi: 10.3389/fpsyt.2021.728204 34658963 PMC8511431

[48] Lu Z Sun Y Zhang Y Chen Y Guo L Liao Y. Pharmacological treatment strategies for antipsychotic-induced hyperprolactinemia: a systematic review and network meta-analysis. Trans Psychiatry. (2022) 12:267. doi: 10.1038/s41398-022-02027-4 PMC9256633 35790713

